# A Centrosaurine (Dinosauria: Ceratopsia) from the Aguja Formation (Late Campanian) of Northern Coahuila, Mexico

**DOI:** 10.1371/journal.pone.0150529

**Published:** 2016-04-13

**Authors:** Héctor E. Rivera-Sylva, Brandon P. Hedrick, Peter Dodson

**Affiliations:** 1 Departamento de Paleontología Museo del Desierto Carlos Abedrop Dávila 3745 Parque Las Maravillas Saltillo, C.P. 25022 Coahuila, México; 2 Department of Earth and Environmental Science, University of Pennsylvania, PA, 19104, United States of America; 3 School of Veterinary Medicine, University of Pennsylvania, PA, 19104, United States of America; Raymond M. Alf Museum of Paleontology, UNITED STATES

## Abstract

While centrosaurines and ceratopsids in general are abundant in the Late Campanian of northern Laramidia, they are much less commonly found in southern Laramidia. This has supported hypotheses of dinosaur provinciality and endemism in the Late Cretaceous with the delineation of at least two separate faunal zones, north and south Laramidia. There have been 12 genera of centrosaurines recognized from northern Laramidia while two genera, *Diabloceratops* and *Nasutoceratops*, have been named from southern Laramidia. We present an osteological description and taphonomic outline for a new centrosaurine ceratopsid from the Aguja Formation of northern Coahuila, Mexico that is not currently diagnosable to the generic level, but likely represents a new taxon. Further, we have included three-dimensional surface scans of all material attributed to this animal. Considering the large number of centrosaurines from northern Laramidia, it is likely that cladistic analyses are biased towards this faunal zone. New findings of southern centrosaurines are needed to correct this bias. This discovery expands the range of centrosaurines south to Coahuila, Mexico and adds new information to better characterize the morphology and taxonomy of centrosaurines from southern Laramidia and their evolution in comparison to their northern counterparts.

## Introduction

The first named ceratopsian dinosaur from Mexico was published in 2010 [1]. In spite of the fact that diagnosable ceratopsian material is seemingly rare in Mexico, the first Mexican ceratopsians were discovered more than fifty years ago. In 1958, several dinosaur specimens were collected in the Parras Basin close to the town of Hipólito, Coahuila. These specimens were identified as belonging to ‘*Monoclonius*’ [2]. During the 1960s, more ceratopsian remains were collected in the Olmos Formation near Palau, northern Coahuila, and were tentatively referred to *Chasmosaurus* [3]. In 1984, a multinational team from the Royal Ontario Museum collected dinosaur bones at Presa de San Antonio, Coahuila. Edmund [4] mentioned that some of the “bones suggest the presence of a ceratopsian”, but did not include figures or further documentation. Lucas and González-Léon [5] reported and figured two vertebral centra of a ceratopsid, which were collected in the Lomas Coloradas Formation and the Corral de Enmedio Formation in Sonora, Mexico; this material was undiagnostic beyond Ceratopsidae. In 2002–2003, a joint team from the Museo del Desierto, the Comisión Paleontológica de Coahuila, and the University of Utah recovered fossil remains, which were referable to both Chasmosaurinae and Centrosaurinae in the Cerro del Pueblo Formation in the Parras Basin of southern Coahuila. In 2010, the first Mexican ceratopsid, the chasmosaurine *Coahuilaceratops magnacuerna*, was described based on skeletal elements of a large adult individual [1].

In spite of a dearth of diagnosable dinosaur discoveries in Mexico for much of the twentieth century, continued interest in Late Cretaceous dinosaur paleontology in Mexico has resulted in a number of recent discoveries, not only from the Cerro del Pueblo Formation [6–10], but also from the Aguja Formation in northwestern Coahuila [11–13]. In addition to body fossils, dinosaur tracks have been discovered across Mexico [14]. The discoveries of *Velafrons*, *Coahuilaceratops*, and *Latirhinus* have revealed that the Cerro del Pueblo Formation has a substantial diversity of dinosaurs [1, 15, 16]. The Aguja Formation in northern Coahuila also has the potential to increase the diversity of Mexican dinosaurs and grant better understanding of Late Cretaceous southern Laramidian faunas.

During consecutive field seasons (2007–2011) in Coahuila, a team from the Museo del Desierto and the University of Pennsylvania discovered a large quantity of vertebrate material representing a variety of taxa, including a substantial percentage of a centrosaurine dinosaur, a pachycephalosaurid tooth, two large hadrosaurid tibiae, numerous crocodilian teeth, and trionychid and baenid turtle shell fragments.

**Fig 1:**
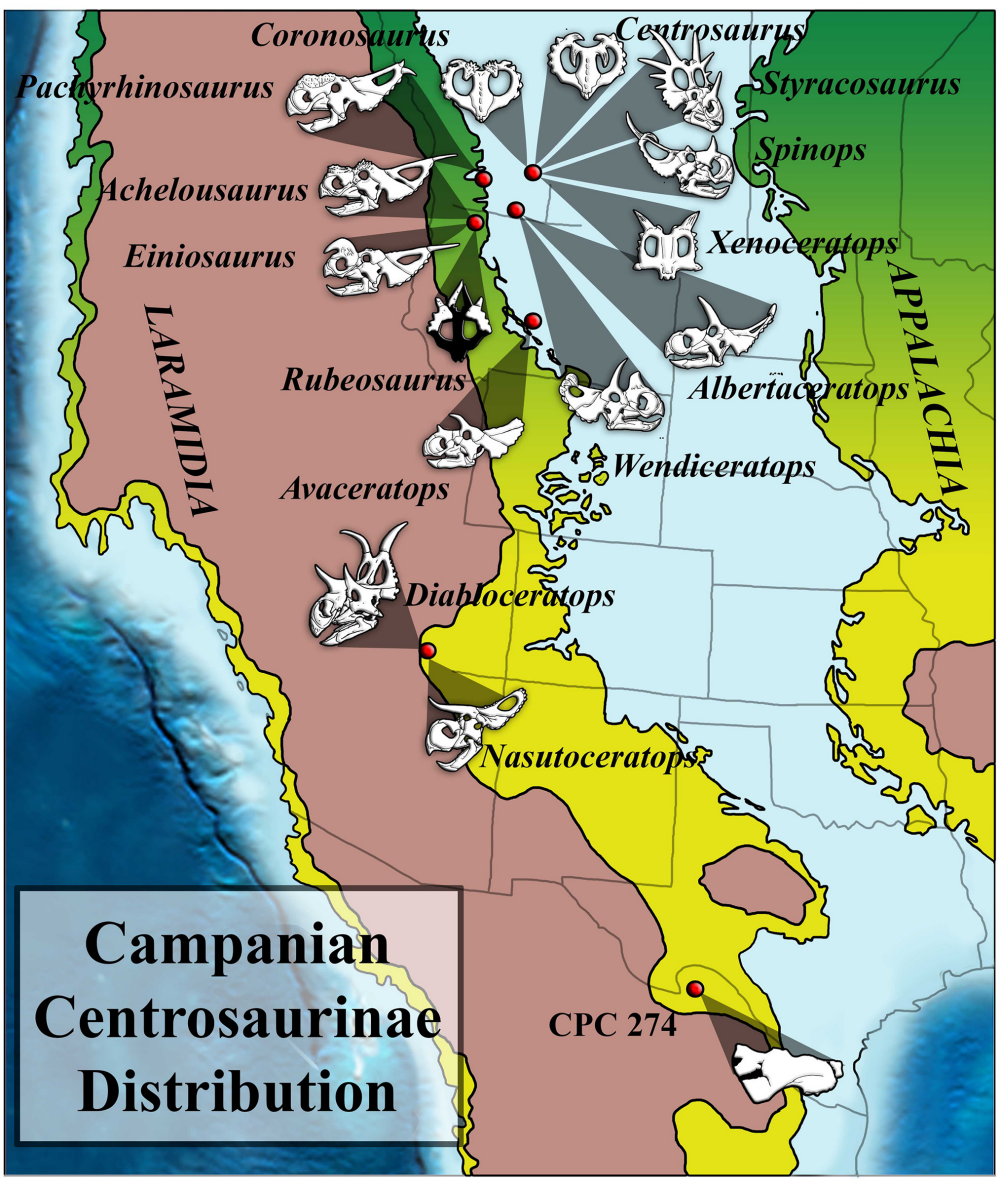
Centrosaurine Biogeography- The Campanian centrosaurine distribution showing that the vast majority of diagnosable Campanian centrosaurines are from northern Laramidia and that the record of southern centrosaurines is sparse. Figure based on Sampson et al. [63]. Individual centrosaurine drawings were redrawn, but based on Brown and Schlaikjer [64] (*Styracosaurus*), Langston [65] (*Pachyrhinosaurus*), Sampson [66] (*Einiosaurus*, *Achelousaurus*), Penkalski and Dodson [46] (*Avaceratops*), Ryan and Russell [67] (*Coronosaurus*, *Centrosaurus*), Ryan [47] (*Albertaceratops*), Kirkland and DeBlieux [20] (*Diabloceratops*), McDonald and Horner [68] (*Rubeosaurus*), Farke et al. [48] (*Spinops*), Ryan et al. [69] (*Xenoceratops*), Sampson et al. [21] (*Nasutoceratops*), and Evans and Ryan [36] (*Wendiceratops*).

Mexican dinosaur taxa (as well as late Campanian taxa from southern Laramidia in general) differ from those of northern Laramidia in both morphology and the dominance of particular dinosaur groups. The late Campanian in northern Laramidia is known from a high diversity of centrosaurines and lambeosaurines, while hadrosaurines and chasmosaurines dominate in southern Laramidia [17–19]. Given the rapid species turnover and limited geographical ranges reconstructed for ceratopsians based on high resolution stratigraphy and excellent material from northern Laramidia [19], it appears highly likely that the diversity known for southern Laramidia in the late Campanian to date may represent a small subset of the diversity that once existed. Centrosaurines in particular are extremely diverse in northern Laramidia, but in southern Laramidia *Diabloceratops* and *Nasutoceratops* are the only known diagnosable centrosaurines (Fig 1) [20, 21]. However, currently undiagnostic centrosaurine material is known from the Menefee Formation [22], the Fort Crittenden Formation [23], and the Cerro del Pueblo Formation [1]. Here, we present a new centrosaurine ceratopsid from Mexico (CPC 274). We show that CPC 274 displays the characters of a basal centrosaurine and we discuss ceratopsian distribution and endemism across Laramidia. Unfortunately this material is not diagnosable to the genus level due to shared apomorphies with other southern Laramidian centrosaurine forms from the Menefee Formation and Fort Crittendon Formation, but the potential discovery of new material of this taxon will likely demonstrate that it is a separate genus from those currently known.

## Geology

**Fig 2:**
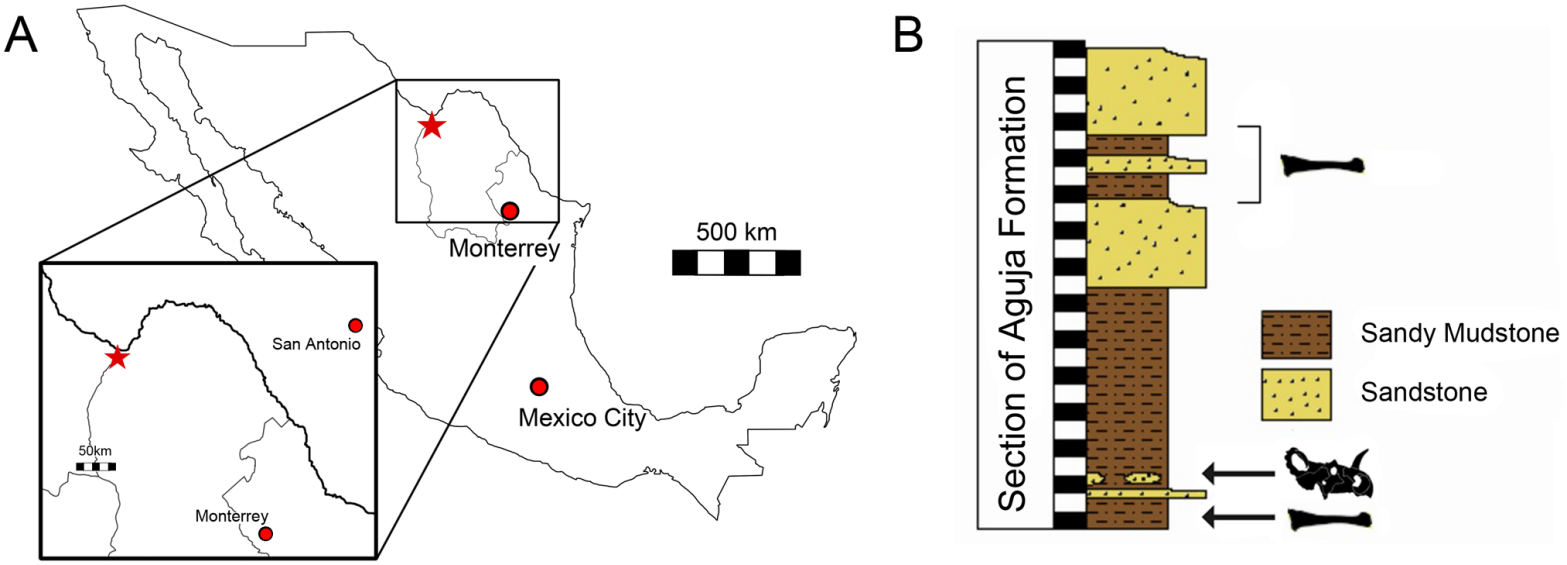
Stratigraphic column of CPC 274- CPC 274 was collected in a section mapped as the Aguja Formation. Unfortunately it was not possible to correlate this section to a section that had either contact with the overlying or underlying formations. CPC 274 is represented by the *Centrosaurus* skull. The two hadrosaur tibiae found at the site are represented as tibiae. 1 box = 1 meter.

The site producing CPC 274 is located in northwestern Coahuila near the town of La Salada in the Aguja Formation [12] (Fig 2). The Aguja Formation is widespread in Big Bend National Park of Texas, where a diverse but generally somewhat fragmentary dinosaur fauna that includes theropods, hadrosaurids, chasmosaurine ceratopsids and pachycephalosaurids [24, 25] is found. The site is located about 23.3 km south of Big Bend, and outcrops of the Aguja Formation are roughly continuous across the Rio Grande. Lithology, sedimentology, and geomorphology confirm the presence of a series of depositional strata consisting of alternating sandstone and sandy mudstone facies with a diverse vertebrate assemblage including dinosaurs, turtles, and crocodilians. The freshwater to brackish fluvial system was situated on a coastal plain, which drained as a delta into the Paleogulf of Mexico [6, 26]; in fact, the site is close to the inferred late Campanian shoreline. The lower part of the Aguja Formation is composed of marine to paralic sandstones and mudstones, and the upper part consists of a prograding clastic wedge [6, 26]. The lower part of the upper member documents deltaic coastal marsh and coastal floodplain environments in which CPC 274 occurs. The sediments were deposited as a deltaic system with a narrow prodelta with marshes, oxbows, and near shore marine deposits [27, 28]. The rest of the upper member consists of floodplain shales with paleosols. Future surveys will yield more material for a detailed reconstruction of the local habitat types within the alluvial system as well as the extent of the system both geographically and temporally.

The area where vertebrate fossils occur is marked tectonically by extension anticlines combined with wide synclines. Both structures are affected by normal faults and lateral faults. Due to a lateral fault in the area, there is a discordant contact between a marine sequence of limestone and mudstones with lamellibranchs and abundant foraminifers that correspond to the marine member of the Aguja Formation. The top of this member follows a transitional sequence of sandstones, mudstones, and limonites, which correlate with the continental sequence of the Aguja Formation. The vertebrate remains occur in these layers. Biostratigraphic correlation using marine invertebrates suggests a late Campanian age for the lower member of the Aguja Formation. According to the stratigraphic data, the new locality in northern Coahuila correlates with the mid to late Campanian. U-Pb SHRIMP-RG analyses of zircon grains from the upper Aguja Formation in Big Bend have been dated to 72.6 Ma +/- 1.5 Ma and 76.9 Ma +/-1.2 Ma [26, 29], and the age of the bottom of the formation is estimated at 80.5 to 81 Ma [30, 31]. There is no sign in our area of the Javelina Formation, which overlies the Aguja Formation in the Big Bend. The fluvio-lacustrine Javelina Formation gives a U-Pb date of 69.0 +/- 0.9 Ma [32] and contains the distinctive *Alamosaurus-Tyannosaurus* fauna, as well as the chasmosaurine *Bravoceratops* [33]. Farther south in the Parras Basin of southern Coahuila is the 4000 m thick Difunta Group that documents an alternating sequence of shallow marine and terrestrial beds. The Cerro del Pueblo Formation at the base of the Difunta group, bears calcareous paludal sediments that contain an important dinosaur fauna dominated by hadrosaurs, including the lambeosaurine *Velafrons coahuilensis* [15] and the saurolophine *Latirhinus uitstlani* [16]. A rare faunal member is the first Mexican ceratopsid, the chasmosaurine *Coahuilaceratops magnacuerna* [1]. The Difunta Group is well dated by both marine invertebrates and by magnetostratigraphy [6, 34] and gives a late Campanian age of 72.5 to 71.5 Ma. Thus the Cerro del Pueblo fauna is either similar in age or slightly younger than the Aguja site in northern Coahuila.

The site is located in a wide plain with dark shales discontinuously intercalated with belts of sandstone. We measured 27.2 meters of the section. Unfortunately, as the site was located on a hill in an open plain, the measured section represents the entire exposed outcrop. The CPC 274 remains were located four meters above the bottom of the section (Fig 2). The sediment matrix that CPC 274 was found within is shale with millimeter-thick dark lamination. The layers slope to the northeast and have many fissures and breaks. Two meters below the centrosaurine remnants, a large hadrosaur tibia was discovered [12]. Between the tibia and CPC 274, sandstone occurs on discontinuous belts intercalated with mudstone, and consists of rounded-angular fine-grained carbonate crystals. A similar sandstone is located 10 meters above the bed that produced CPC 274 with a thickness of approximately four meters. A second large hadrosaur tibia (> 1 meter in length) was found above this sandstone between meters 19–22 (Fig 2). Unfortunately, the exact location in the section was not recorded.

## Materials and Methods

The fossils that form the subject of this report were collected during field seasons of 2007 to 2011. All materials are catalogued in the collections of the Museo del Desierto in Saltillo, Coahuila, Mexico. Measurements of the CPC 274 and CPC 1478 material were taken using soft measuring tape for elements longer than 300 mm and Mitutoyo 500–173 digital calipers for elements shorter than 300 mm (Tables 1–9). Measurements reported in the manuscript were taken between perpendicular lines parallel to the long axis of the bones. In addition to photographs and traditional measurements, all identifiable bones were surface scanned using a Polhemus FastSCAN system in order to capture three-dimensional geometry. Scans were post-processed using Geomagic^™^. 3D surface scans of all bones are available in the supplemental information (S1–S8 Multimedia) and are reposited on the website repository.upenn.edu/vp3d. TNT (Tree analysis using New Technology) [35] was used for the cladistic analysis. To assess the systematic position of CPC 274, it was coded into the character dataset generated by Evans and Ryan [36]. The exact coding for CPC 274 is found in the supplement (S1 Table). Tree statistics (consistency index (ci), retention index (ri)) and Bremer support values were calculated using TNT [35]. We reported both the strict consensus tree and 50% majority rule trees. Approximate modern latitude values of centrosaurine taxa were entered into Matlab [37] in order to generate a continuous gradation heat-map (using the colormap and imagesc functions) to show the relative latitude of specimens (Fig 1). Cooler colors are higher latitudes and hotter colors are lower latitudes.

**Table 1:** Squamosal Measurements.

Squamosal (CPC 274)	
Rostrocaudal length (preserved)	306 mm
Dorsoventral height (preserved)	177 mm
Rostrocaudal length of frill component of squamosal	249 mm

**Table 2:** Parietal Measurements.

Parietal Fragment (CPC 274)	
Length of frill margin (preserved)	140 mm
Length of central fragment	177 mm
Length of small fragment	66 mm

**Table 3:** Dentary Measurements.

Dentary (CPC 274)	
Length of dentary (preserved)	134 mm
Length of tooth row (preserved)	70 mm
Height of coronoid process (preserved)	70 mm

**Table 4:** Premaxilla Measurements.

Premaxilla (CPC 274)	
Rostrocaudal length (preserved)	92 mm
Dorsoventral height (preserved)	61 mm

**Table 5:** Scapula Measurements.

Scapula (CPC 274)	
Craniocaudal length	464 mm
Height of acromion process	133 mm
Proximal dorsoventral height	114 mm
Distal dorsoventral height	109 mm
Length of glenoid	80 mm

**Table 6:** Ilium Measurements.

Preacetabular Process of Ilium (CPC 274)	
Craniocaudal length (preserved)	296 mm
Dorsoventral height (preserved)	74 mm

**Table 7:** Femur Measurements.

Femur (CPC 274)	
Proximodistal length	549 mm
Length to fourth trochanter	253 mm
Width of femoral head	199 mm
Width of distal condyles	136 mm
Midshaft circumference	26 mm

**Table 8:** Dorsal Vertebra Measurements.

Dorsal Vertebra (CPC 274)	
Bilateral width of centrum	73 mm
Dorsoventral height of centrum	80 mm
Craniocaudal length of centrum	51 mm
Height of neural arch (preserved)	52 mm
Width of neural arch (preserved)	67 mm
Neural canal dorsoventral height	36 mm
Neural canal bilateral width	26 mm

**Table 9:** Tibia Measurements.

Tibia (CPC 1478)	
Width of proximal condyles	60 mm
Proximodistal length (preserved)	98 mm
Mediolateral width	100 mm

### Permits

All materials were collected with permission from the Instituto Nacional Antropología e Historia National through its National Council of Paleontology under permit number 401.13–352.

Systematic Paleontology:

DINOSAURIA [38]

ORNITHISCHIA [39]

CERATOPSIA [40]

NEOCERATOPSIA [41]

CERATOPSIDAE [42]

CENTROSAURINAE [43]

Material––CPC 274 is an indeterminant centrosaurine ceratopsid composed of a partial skull and partial postcranium consisting of a nearly complete right squamosal, fragmentary parietal, dentary, premaxillary fragment, complete scapula and femur, left preacetabular process of the ilium, and partial dorsal vertebra. It is not yet clear where in the Aguja Formation the locality occurs due to the low relief leading to a lack of nearby rocks that can be correlated with the field site. The sediments are consistent with the basal portion of the upper member of the Aguja, documenting the transition from marine to terrestrial sedimentation. Further work on the geology of the area is required to better understand the exact horizon of the type locality within the Aguja Formation. GPS coordinates are N 28° 46’ 26.8”, W 103° 19’ 51.7”.

## Results

### Osteological Description

#### Squamosal

**Fig 3:**
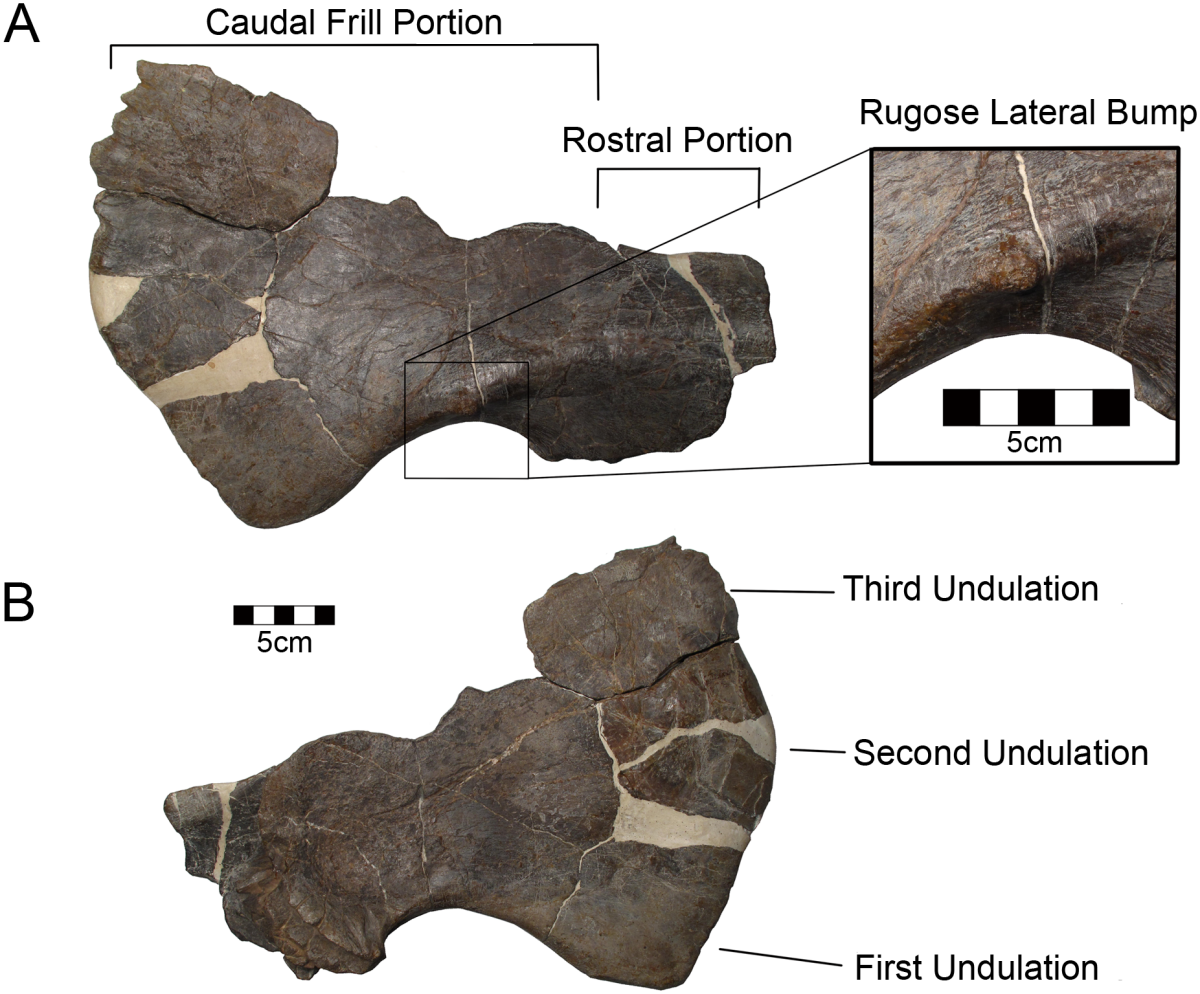
CPC 274 Squamosal- The right squamosal of CPC 274 in (A) lateral and (B) medial views. Scale = 5 cm. Inset showing the rugose lateral bump. Scale = 5 cm.

The majority of the right squamosal including the entire free border of the squamosal part of the frill is well preserved (Fig 3; Table 1). The squamosal demonstrates the general shape of the centrosaurine squamosal including the characteristic step contact with the parietal [44]. The shape of the squamosal differs somewhat from *Styracosaurus*, *Centrosaurus*, and *Pachyrhinosaurus* [45] in that it is longer rostrocaudally than wide, similar to *Avaceratops* [46]. The slot for the jugal is well formed and the articular surfaces for the quadrate and paroccipital process are complex. Medially, the groove for the paroccipital separates rostral and caudal portions in the squamosal as in other centrosaurines. The caudal portion is twice as long as the rostral portion as in centrosaurines other than *Avaceratops*. The quadrate process is broken.

Along the ventral margin of the frill, there is a crescentic postquadrate curve or embayment expanding caudally to the ventral scallop or undulation as in other centrosaurines. Three broad scallops are seen along the caudal edge of the squamosal but they are not strongly developed as they are in *Wendiceratops*, which has four [36]. The second scallop is dorsally deflected in comparison with the lateral scallop similar to *Albertaceratops*, *Spinops*, *Centrosaurus*, and *Styracosaurus* [45, 47, 48]. Larger centrosaurines such as *Styracosaurus*, *Centrosaurus*, *Pachyrhinosaurus*, and *Einiosaurus* have four or five squamosal scallops. The caudodorsal border of the squamosal is thickened (18 mm) where it contacts the parietal.

The three lateral bosses present on some centrosaurine squamosals (*Avaceratops*, *Albertaceratops*, *Wendiceratops*) are not evident, though Ryan [47] notes that these are of variable development in Centrosaurinae. The ridge is very strong in *Nasutoceratops* and runs most of the length of the squamosal. Sampson et al. [21] report that a similar feature is found in several centrosaurine squamosals, including one from the late Campanian Fort Crittenden Formation of Arizona [NMMNH P-34906; 23] and one from the early Campanian Menefee Formation of New Mexico [NMMNH P-25052; 22]. In CPC 274 the ridge is more modest and ventral in position compared to *Nasutoceratops* and does not extend onto the expanded fan of the caudal portion of the squamosal; instead it ends over the postquadrate embayment. The ridge is also short in the Menefee squamosal. There is a small rugose bump on the lateral aspect of the squamosal just above the ventral crescentic margin. This feature likewise appears on the Menefee squamosal [22]. However, the bump is less developed. If these two structures are homologous, this bump may be partially resorbed through ontogeny or varies in development among closely related taxa. Other than NMMNH P-25052, this feature is not known within Centrosaurinae. The small size and apparent rugosity precludes its homology with the three lateral bumps seen on other centrosaurines. There are numerous striations extending parallel to the long axis of the squamosal on the lateral aspect similar to other centrosaurines (*Albertaceratops*, NMMNH P-25052). These striations are less pronounced on the medial aspect.

#### Parietal

**Fig 4:**
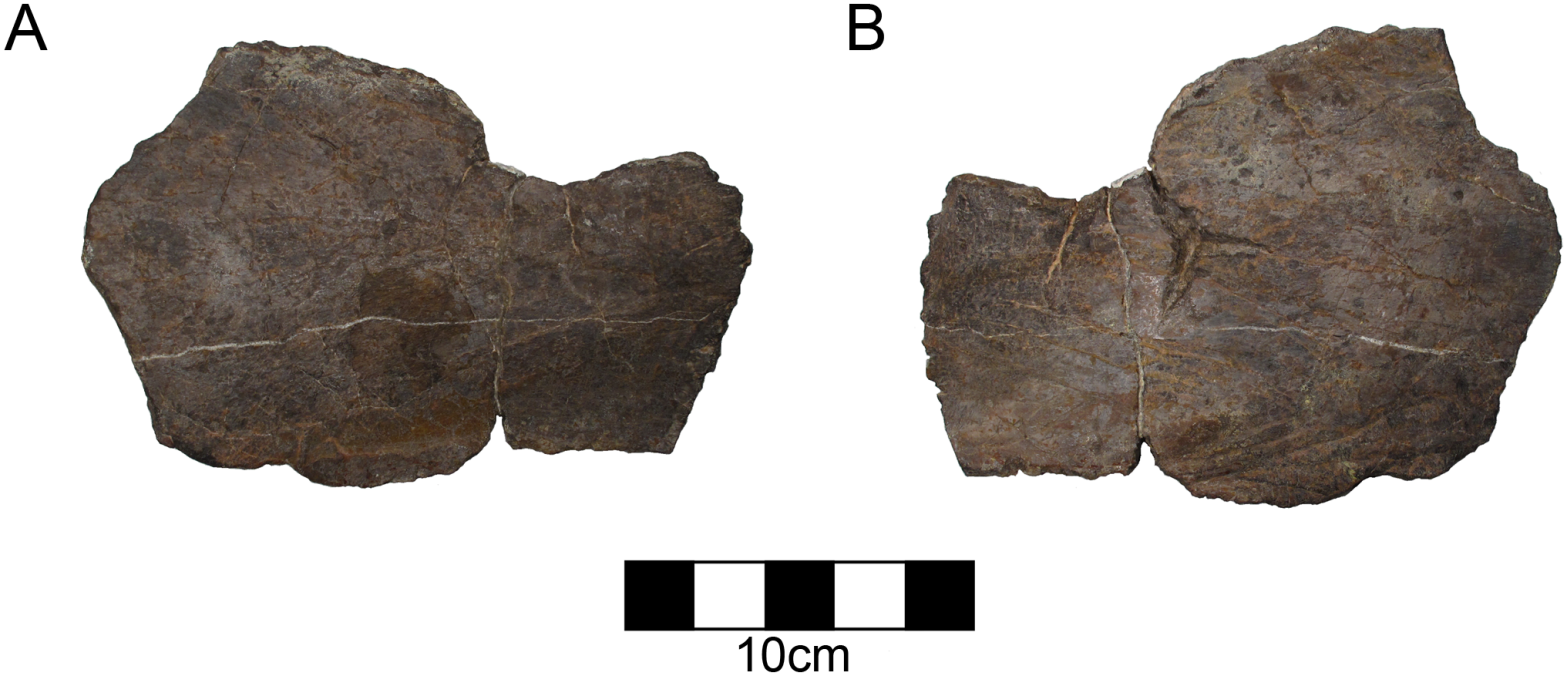
CPC 274 Parietal- The partial fragment of CPC 274 in both front and back views (A, B). No parietal edge is preserved in this fragment. Scale = 10 cm.

**Fig 5:**
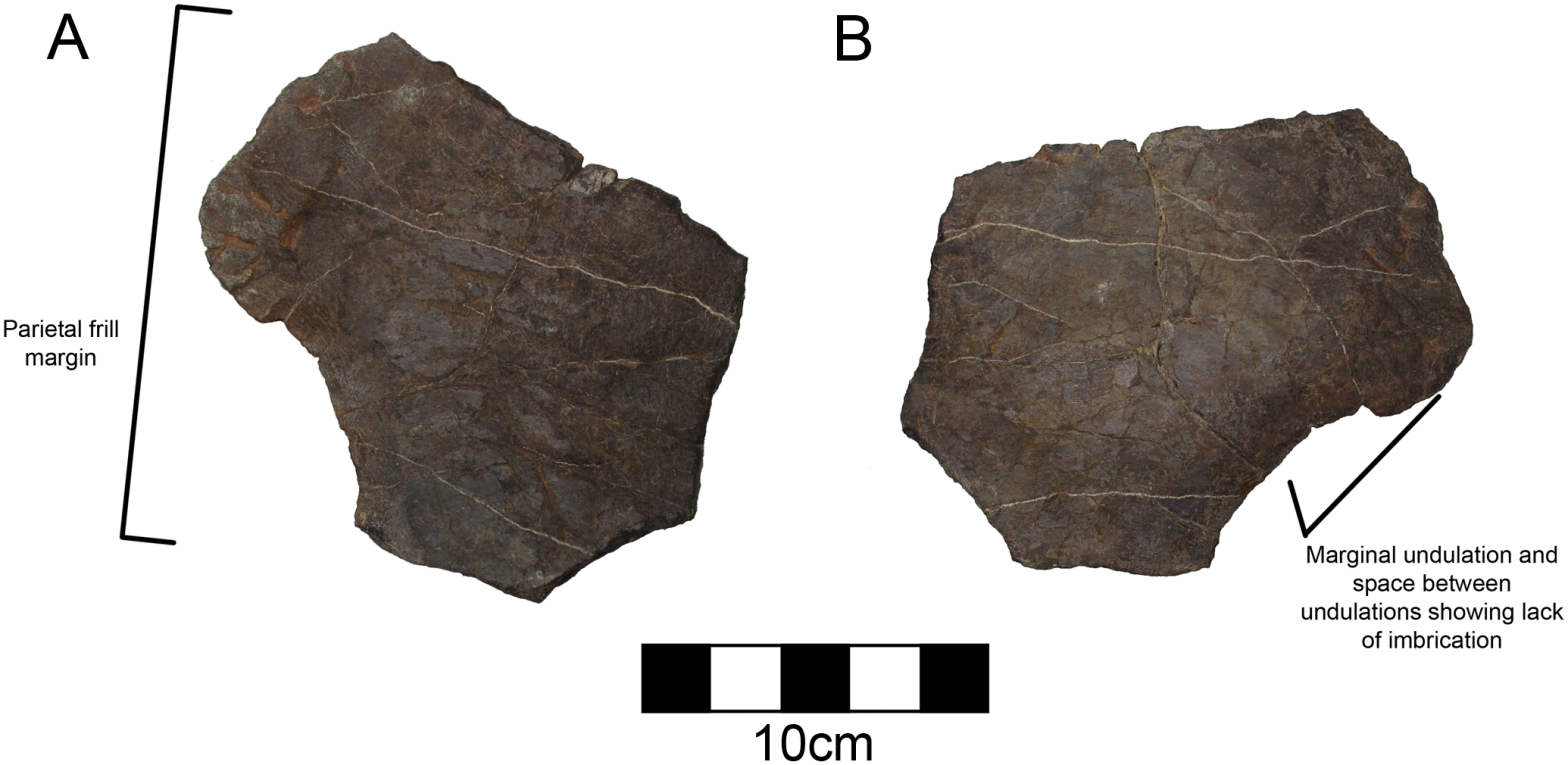
CPC 274 Parietal- One parietal fragment of CPC 274 in both front and back views (A, B) showing one of the parietal undulations. Scale = 10 cm.

There are two broad flat parietal fragments and a smaller fragment from the rostral midline. Together they approximate the size of the squamosal suggesting they compose about one sixth to one fourth of the total parietal portion of the frill. The dorsal and ventral surfaces are comparatively smooth with a few shallow vascular channels in evidence. The largest fragment is a saddle-shaped segment of the middle of the frill that contains no edge for the squamosal, parietal fenestrae, or frill margin (Fig 4). It is 16 mm thick at the thickest point and thins to 6 mm. The second fragment has part of the frill margin and has a single epoccipital (Fig 5; Table 2). It is 14 mm at its thickest along the broken edge, and thins to as little at 3.2 mm along the free edge. The epoccipital is rounded and forms a simple crescent similar to the lateral undulations on centrosaurine squamosals or midline scallops in *Avaceratops* [46]. There is marked rugosity along it contrasting with the smoother dorsal surface of the parietal. The epoccipital is firmly fused to the parietal, but the suture has not been obliterated on the ventral surface. To the side of the scallop, there is a concave embayment suggesting undulations between convex scallops, but the undulations are not imbricated. The concave embayment is slightly larger than the scallop itself. The surface of the bone is well vascularized on the dorsal aspect and has many vascular grooves. It is 14.9 mm thick at is base but thins to 5.0 mm at its apex.

Both of the larger pieces are curved along their surfaces and are thickened in cross section suggesting that neither of the edges approached the parietal fenestrae, if such fenestrae were present. The overall size of the bone surface and the modesty of thinning towards the parietal fenestrae preclude the potential for a parietal with large parietal fenestrae as in other centrosaurines (*Albertaceratops*, *Diabloceratops*, *Xenoceratops*, *Nasutoceratops*, *Wendiceratops*, *Centrosaurus*, *Styracosaurus*, *Einiosaurus*, *Achelousaurus*, *Pachyrhinosaurus*) and the discovery of more complete material may demonstrate that there were no parietal fenestrae in this taxon. The small parietal fenestrae (or complete lack of fenestrae) in the holotype of *Avaceratops* (ANS 15800) could be a juvenile feature. While there are characteristics of ANS 15800 that are juvenile such as overall size, bone texture, tooth count, and a lack of fused sutures, CPC 274 is either late-subadult or adult based on suture closure as has been demonstrated for crocodilians [49]. Therefore, finding other centrosaurines without parietal fenestrae may demonstrate that the lack of parietal fenestrae is a real character for *Avaceratops*, supported by MOR 692, the larger specimen referred to *Avaceratops* [46]. However, only through future finds will this be shown.

The third portion of parietal represents the rostral end of the parietal close to the point of contact with the frontal. There are numerous sinus cavities ventrally. This somewhat amorphous fragment measures 65 by 61 mm, and roofs the caudal portion of the frontal fontanelle.

#### Dentary

**Fig 6:**
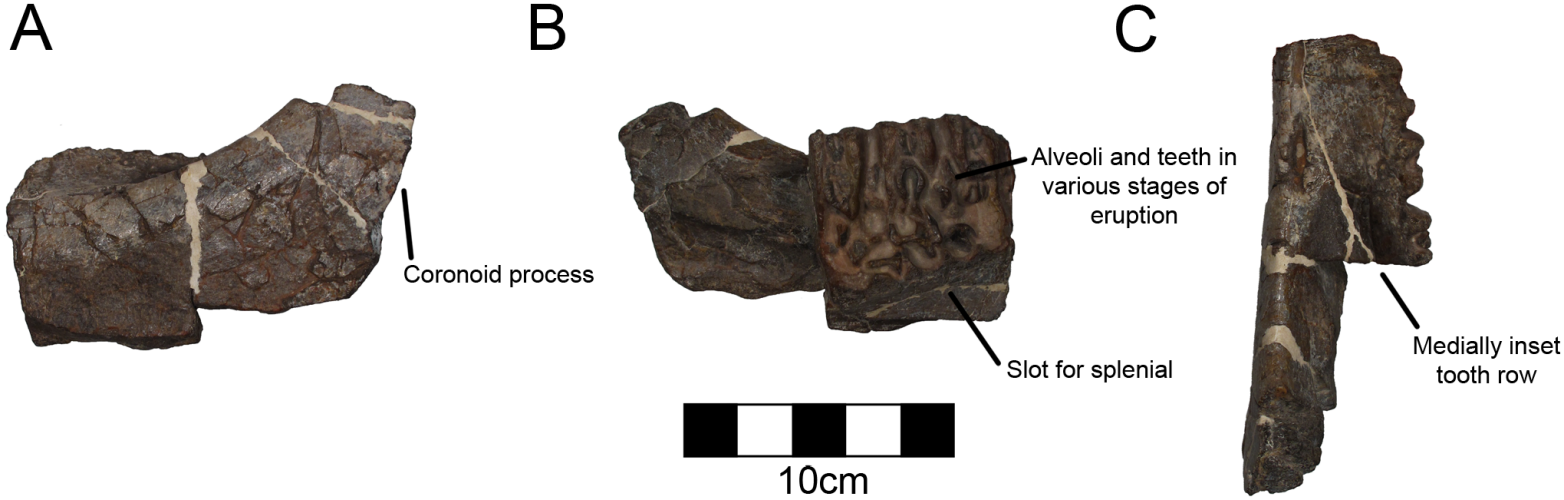
CPC 274 Left Dentary- A fragment of the left dentary of CPC 274 in (A) lateral, (B) medial, and (C) dorsal views. Only a section of the tooth row is preserved and it is assumed based on other centrosaurines that the tooth row extends at least to the back of the coronoid process. Scale = 10 cm.

A caudal fragment of the left dentary is preserved with six tooth sockets containing eleven total teeth in various states of eruption (Fig 6; Table 3). The tooth row is medially inset as in other ceratopsids (Fig 6C). There are a maximum of three teeth preserved in progression. Three teeth are fully erupted. The other three sockets do not preserve an erupted tooth. It would appear that these six tooth sockets are in the caudal third of the tooth row due to the dorsal inclination of the dentary forming the coronoid, which occurs in the caudal third of the tooth row in many other centrosaurines (*Avaceratops*, *Styracosaurus*, *Pachyrhinosaurus*). However, the caudal aspect is broken so this cannot be confirmed. The ventral margin of the dentary is also broken, though there is a deep slot ventrally for the splenial as in other centrosaurines (Fig 6B).

#### Premaxilla

**Fig 7:**
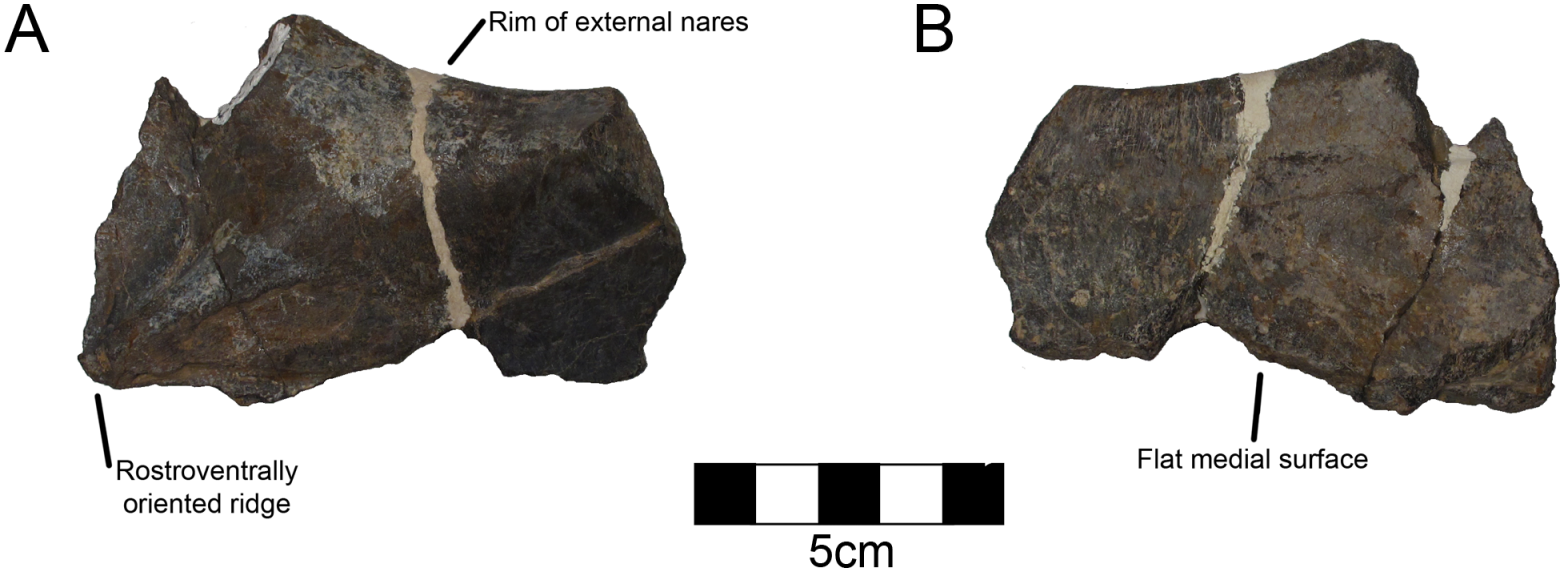
CPC 274 Right Premaxilla- A fragment of the right premaxilla of CPC 274 in (A) lateral and (B) medial views showing the ventral rim of the external nares. Scale = 5 cm.

A fragment of the right premaxilla preserves the ventral rim of the external nares as well as the ridge extending diagonally with a rostroventral orientation (Fig 7; Table 4). The ridge is better defined than in *Avaceratops* [46], but is similar to the structure in other centrosaurines like *Diabloceratops* [20]. The preserved aspect of the bone is thickest along the narial margin. Although the medial aspect of centrosaurine premaxillae is generally complex, the area just ventral to the external nares is smooth. The medial aspect of CPC 274 is completely smooth as in other centrosaurines. There is no evidence for foramina on either the medial or lateral surfaces.

#### Scapula

**Fig 8:**
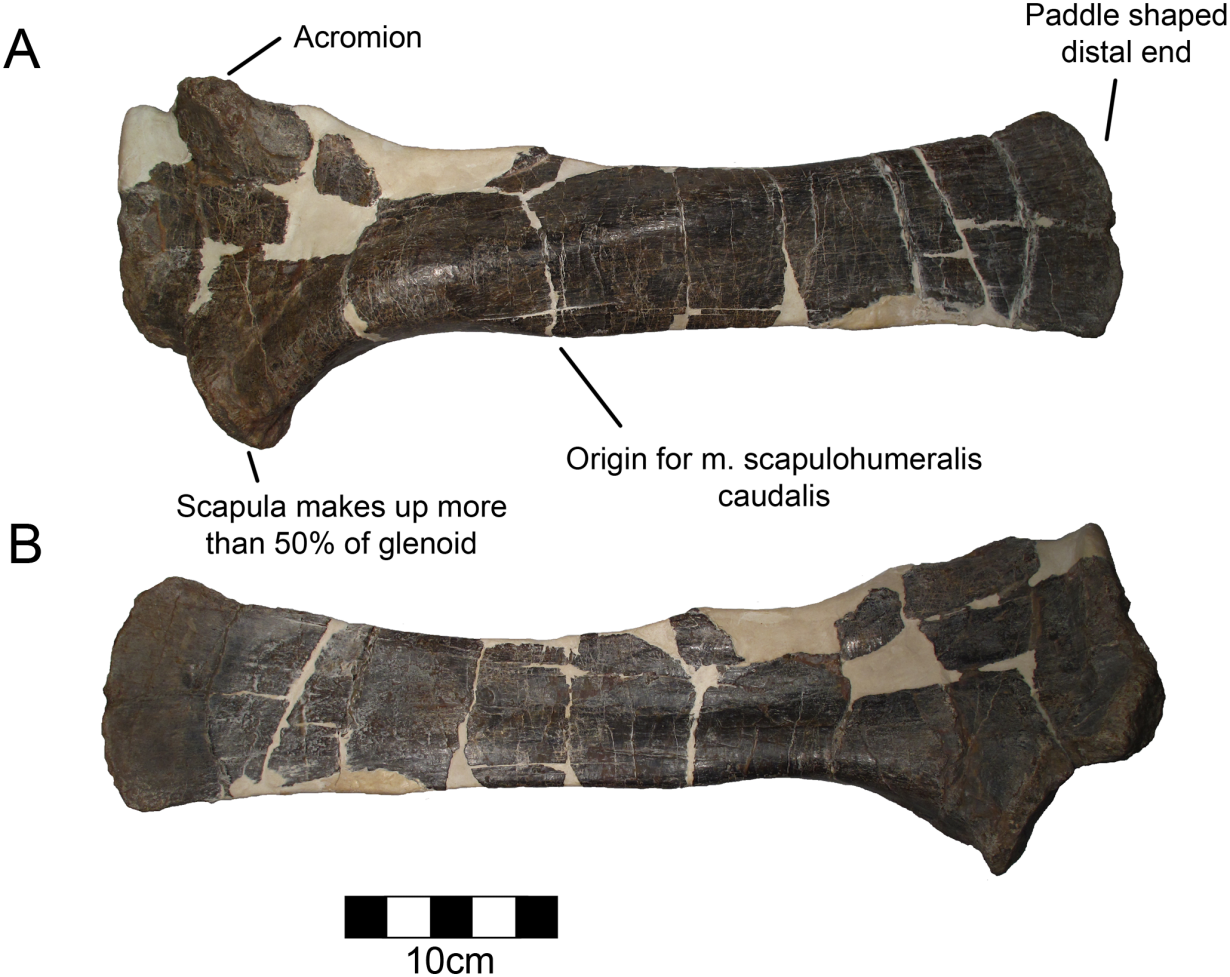
CPC 274 Left Scapula- The complete left scapula of CPC 274 in (A) lateral and (B) medial views. Scale = 10 cm.

The left scapula is preserved and is typically ceratopsid (Fig 8; Table 5) [50, 51]. The scapula contributes more than half of the glenoid as is typical for Centrosaurinae [44]. The articulation for the coracoid is thickened and nearly perpendicular to the long axis of the scapula. There is a well-defined acromion that is highly rugose and laterally projecting as in other ceratopsids. There is a defined rugose point for the m. triceps brachii just caudal to the glenoid. The origin for the m. scapulohumeralis caudalis is caudal to the triceps origin and manifests as a slight downward expansion on the ventral margin of the scapular blade. The scapula is thickened around the curve dorsal to the origin of the m. scapulohumeralis. This is the thickest point of the scapula and was probably thick in order to resist torsion. The blade of the scapula does not angle gently, but it is bent at about 25° at the level of the thickened region. The scapula is straight both proximal and distal to this region rather than curving gradually. This feature does not appear to be diagenetic. The distal scapula is somewhat expanded both dorsally and ventrally forming a paddle shape more evident than in some centrosaurines such as *Centrosaurus* [45]. There is evidence of a cartilaginous suprascapular region attached along the distal blade, as there is marked rugosity. The medial aspect of the scapula is flat, though it is slightly concave opposite of the middle thickening.

#### Ilium

**Fig 9:**
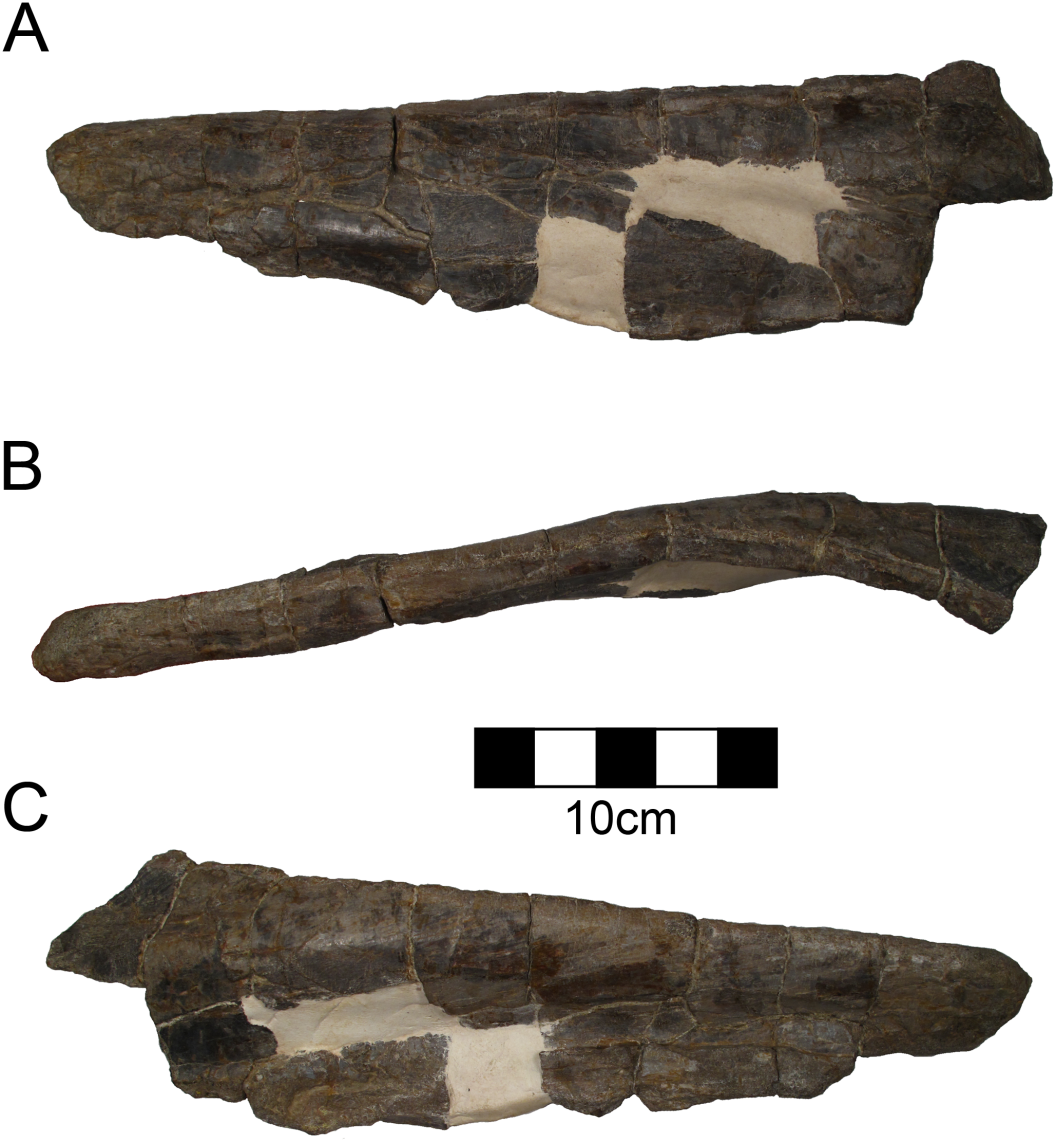
CPC 274 Partial Ilium- The left preacetabular process of the ilium of CPC 274 showing a strong lateral deflection as in other ceratopsians in (A) lateral, (B) dorsal, and (C) medial views. Scale = 10 cm.

The left preacetabular process of the ilium is preserved (Fig 9; Table 6). The end is complete, but the base is incomplete and it is not possible to determine the total length. As preserved, it is 296 mm long. There is proximal curvature towards the body of the ilium. The cranial portion is completely straight and is laterally deflected. Most of the ventral aspect is preserved though it is diagenetically distorted. There is a slight groove running along the proximal portion of the ventral aspect likely for the origin of the m. puboischiofemoralis. The dorsal aspect is medially inset in comparison with the ventral aspect and is flattened.

#### Femur

**Fig 10:**
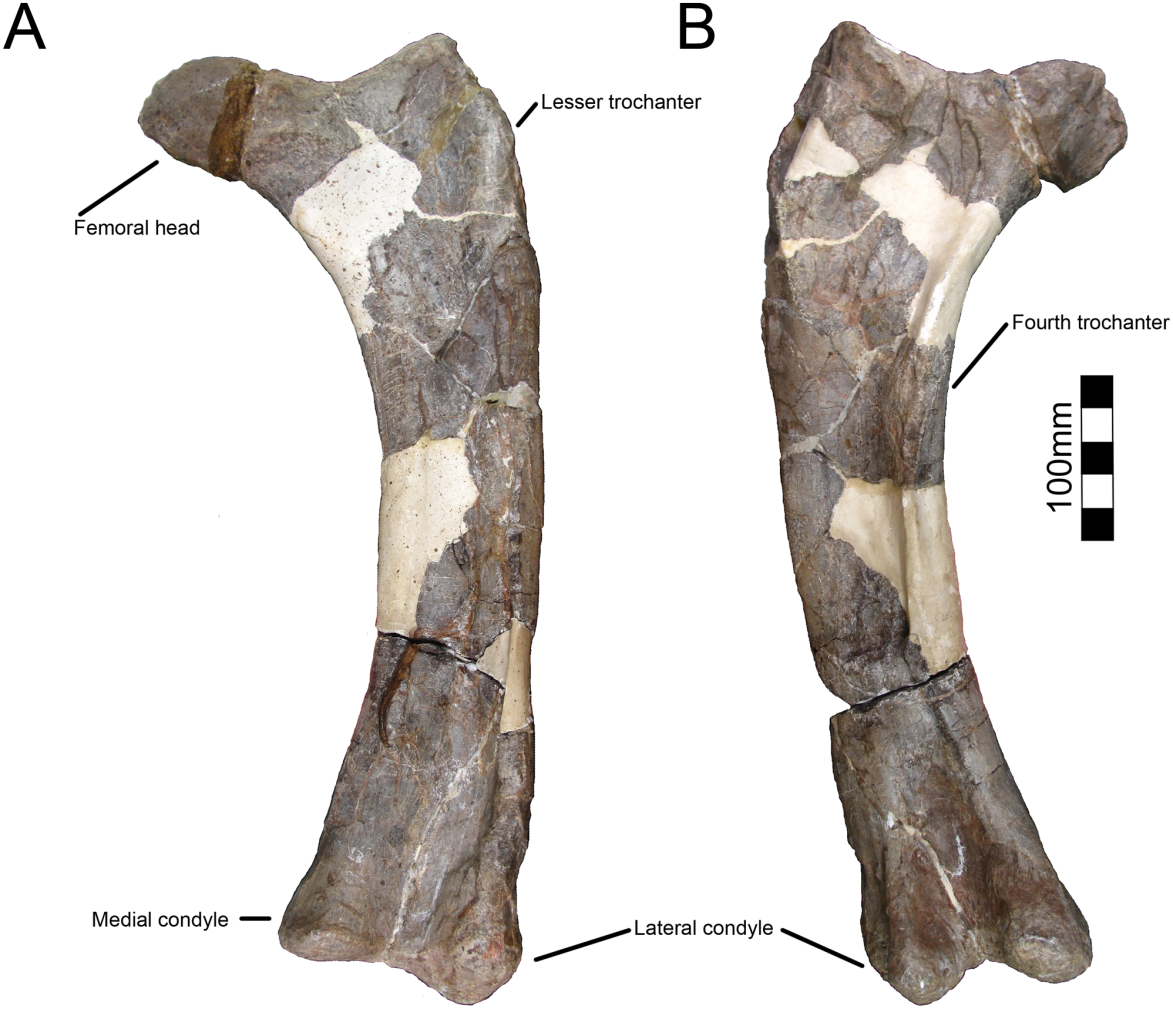
CPC 274 Left Femur- The left femur of CPC 274 in (A) cranial and (B) caudal views. Scale = 10 cm.

The complete left femur is preserved, though it is moderately crushed craniocaudally (Fig 10; Table 7). The femur is straight and has minimal curvature like *Avaceratops* [46], though this may be related to the postmortem distortion. The medially-facing femoral head is well rounded and semi-globose. The greater trochanter is separated from the head of the femur and rises dorsally above the femoral head. The fourth trochanter is located proximal to midshaft and forms a prominent crest-like structure as in other centrosaurines. The femoral shaft, though compacted, is robust.

#### Dorsal Vertebra

**Fig 11:**
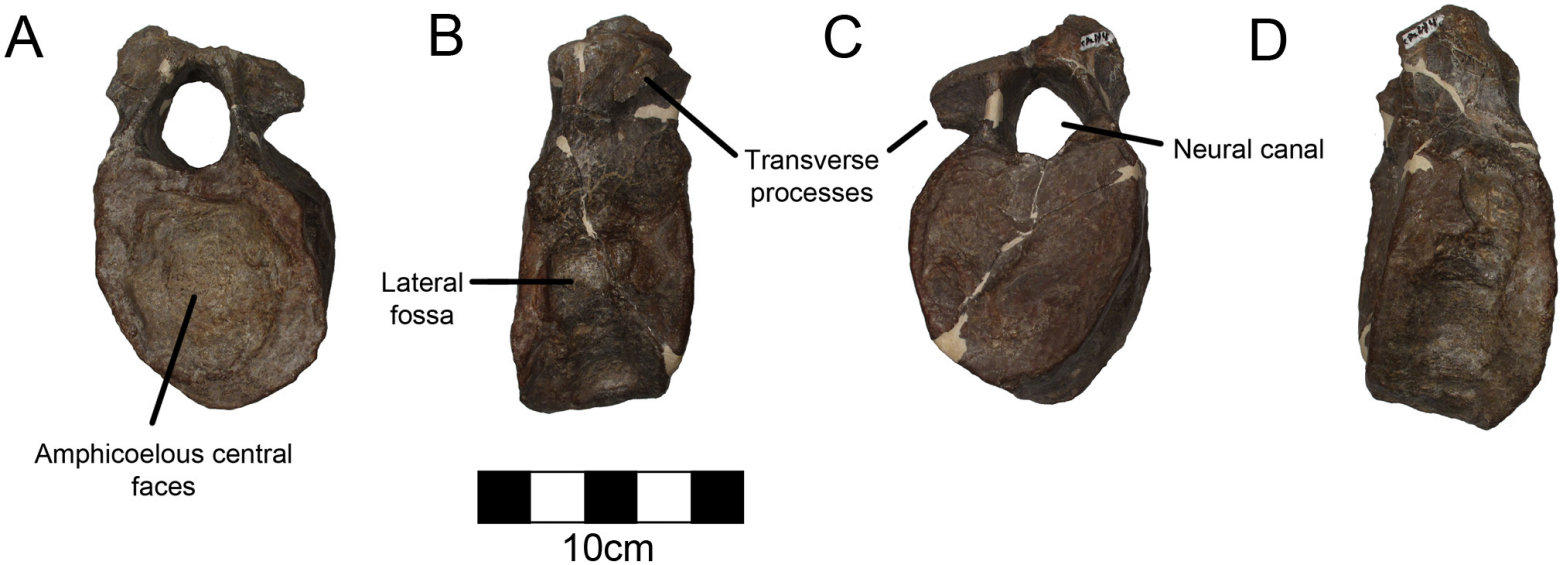
CPC 274 Dorsal Vertebra- A complete dorsal centrum and ventral neural arch in (A) cranial, (B) left lateral, (C) caudal, and (D) right lateral views. Scale = 10 cm.

A dorsal vertebra is preserved including the entirety of the centrum and the ventral part of the vertebral table with a complete neural canal (Fig 11; Table 8). However, the neural spine and transverse processes are broken from the vertebral table. The central faces are strongly amphicoelous as in other ceratopsids and are elliptical with a slight divot just below the neural canal. The neural canal is very large; approximately one-quarter the size of the articular faces of the centrum, as opposed to closer to one sixth in other ceratopsids. This suggests that this is a more cranial dorsal near the cervicodorsal transition where the neural canal is larger in order to accommodate the brachial plexus [52]. This is further supported by the fact that the transverse processes are located above the centrum. The dorsoventral axis of the neural canal is about 25% greater than the horizontal axis. The neural arch is completely fused to the centrum with a trace of the suture just above the centrum. However, this grades to obliterated in other places. The sides of the centrum are pinched in and there is a single fossa on either side just below the neural canal on the lateral aspect.

#### Tibia

**Fig 12:**
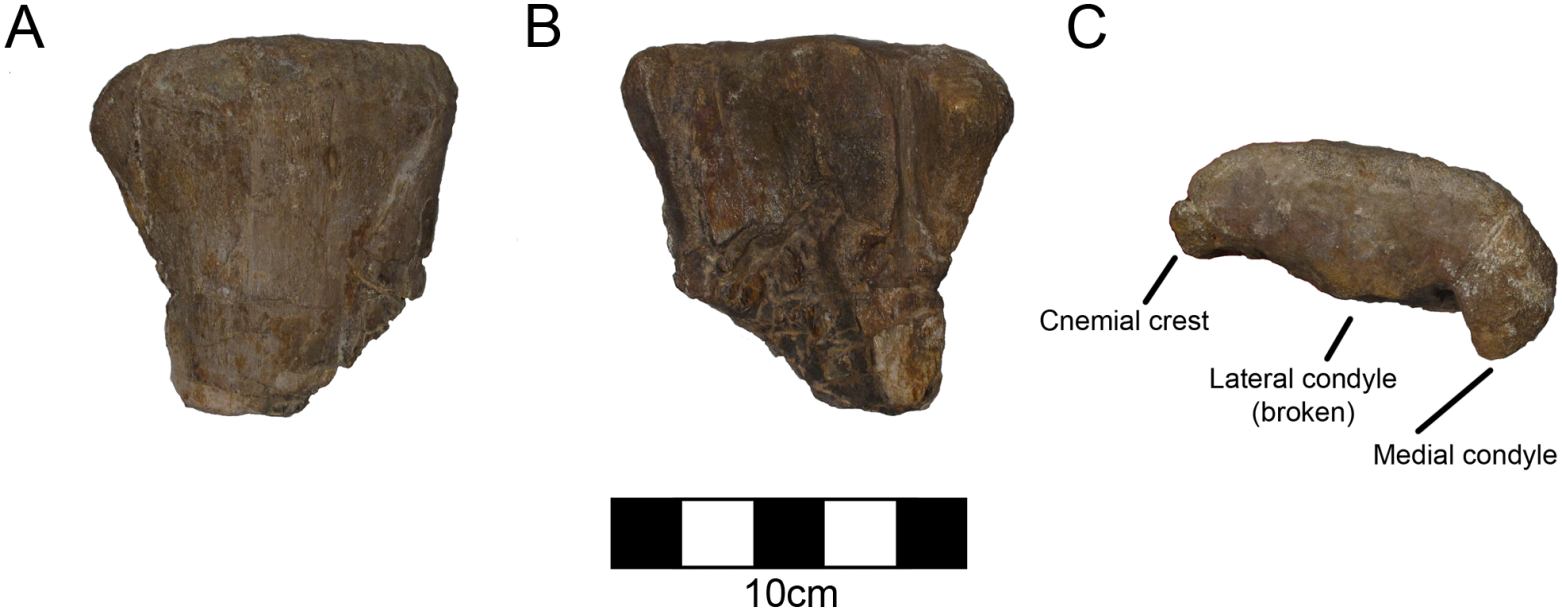
CPC 1478 Left Tibia- The proximal left tibia of another smaller ceratopsian found in the same quarry as CPC 274 in (A) cranial, (B) caudal, and (C) proximal view demonstrating the pronounced cnemial crest and proximal condyles. Scale = 10 cm.

The left proximal tibia of a separate animal (CPC 1478) is preserved with the rest of the ceratopsian material (Fig 12; Table 9). Although it comes from the same quarry as CPC 274, it is too small to be referred to CPC 274 suggesting at least two animals in the quarry. Both proximal condyles as well as the cnemial crest are well preserved, but the distal portion has been lost. The cnemial crest is expanded laterally and is approximately 40% of the width of the condylar part of the tibia. There is abundant hadrosaur material found both above and below the CPC 274 quarry so hadrosaurs were clearly members of this community. The relative size of the cnemial crest is common for ceratopsians and it resembles the size and shape of *Avaceratops* (ANS 15800) [45]. Therefore, this tibia probably belonged to a small ceratopsian rather than a small hadrosaur.

### Phylogenetic Analysis

In order to establish the phylogenetic relationships of CPC 274 with respect to other ceratopsid genera, a phylogenetic analysis was undertaken. Recently Evans and Ryan [36] published a phylogenetic analysis of the Centrosaurinae to accommodate their new taxon, *Wendiceratops pinhornensis*. Due to the lack of material currently characterizing CPC 274, we opted to not name the taxon, but still ran a phylogenetic analysis in order to understand its relationships with known taxa. We placed it into the matrix of Evans and Ryan [36] rather than creating a new matrix. Our analysis included 26 OTUs with the addition of *Wendiceratops pinhornensis* [36] and CPC 274. Of the original 101 characters used by Evans and Ryan [36], it was possible to code only fourteen characters for CPC 274. This is due to the large amount of convergence in the postcranial skeleton of ceratopsids and the lack of a complete parietal in CPC 274.

**Fig 13:**
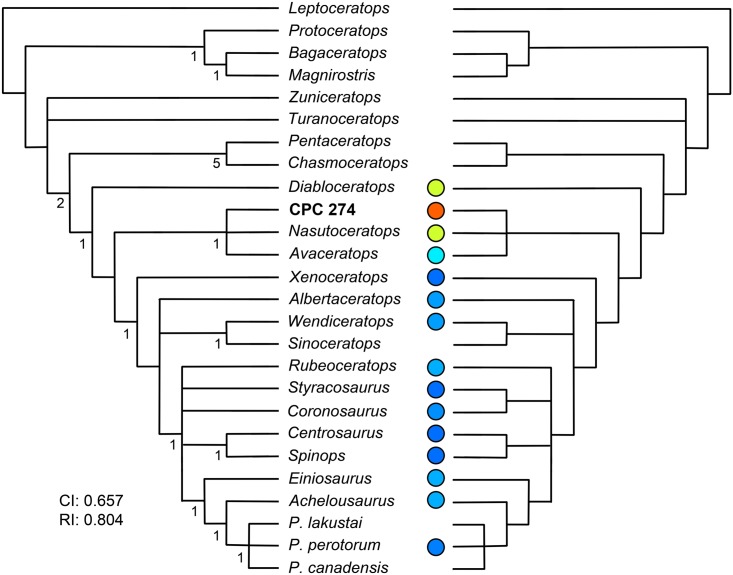
Cladogram- (A) The strict consensus tree and (B) 50% majority rule tree placing CPC 274 in the matrix of Evans and Ryan [36]. Colored circles show approximate latitudes where specimens were discovered. Hotter colors indicate lower latitudes.

We ran strict consensus analyses and 50% majority rule analyses (Fig 13; S1 Table) and generated a consistency index of 0.657 and retention index of 0.804. Both analyses placed CPC 274 in a basal polytomy with the basal centrosaurines, *Avaceratops* and *Nasutoceratops* (Fig 13). This clade was more derived than *Diabloceratops*, but less so than *Xenoceratops*.

## Discussion

### Phylogeny and Relationships with other Taxa

Our phylogenetic analysis recovers CPC 274 as a basal centrosaurine in a polytomy with the basal centrosaurines, *Avaceratops* and *Nasutoceratops*. *Diabloceratops* is found to be basal to this clade. *Diabloceratops* has many plesiomorphic characters that are very different from more derived centrosaurines resulting in its basal position [20]. *Diabloceratops* is also the oldest known centrosaurine at 79.5 Mya. *Diabloceratops* and mid-Campanian *Nasutoceratops* are the only centrosaurines included in the analyses from southern Laramidia [20]. CPC 274 is from the late Campanian, when centrosaurines were seemingly much more common in northern Laramidia. It is possible that CPC 274 is grouping with basal centrosaurines including *Nasutoceratops* because of geographic association and is not actually a basal form itself. However, its sharing several characters with *Avaceratops*, which also frequently comes out as a basal form in analyses in which it is included [21, 36, 48] suggests that CPC 274 may also be basal and the phylogenetic result is not based only on geographic association. Further finds of more material of CPC 274, especially more parietal material, and the discovery and inclusion in cladistic analyses of more southern centrosaurine forms will be necessary in order to better unravel its phylogenetic position.

**Fig 14:**
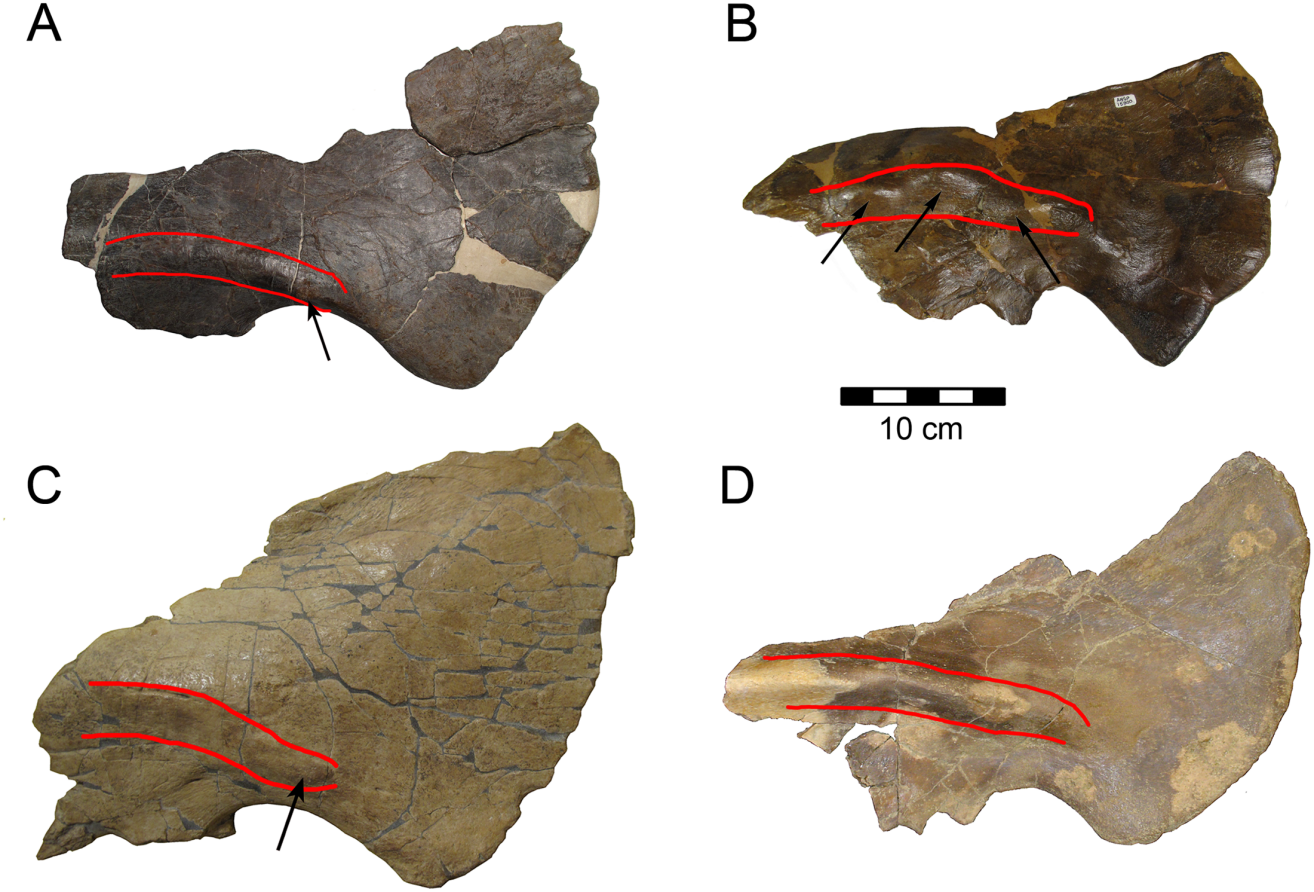
Squamosal Comparison- (A) CPC 274, (B) *Avaceratops*, (C) NMMNH P-25052, and (D) *Nasutoceratops*. Red lines indicate the raised ridge that runs along each squamosal with black arrows pointing at prominences.

There are two notable synapomorphies of CPC 274, the short ventrally- placed oblique ridge on the squamosal and the robust knob along the ridge, which are shared with NMMNH P-25052 from the Menefee Formation of New Mexico [22] (Fig 14), and possibly with NMMNH P34906 from the Fort Crittenden Formation. However, these features are not seen on northern centrosaurines. Another centrosaurine to mention is contemporary with *Diabloceratops* and is represented by a partial skull from the Wahweap Formation. This skull, UMNH VP16704, is known as the Nipple Butte skull [20, 53]. The well preserved right squamosal shows the fan-shaped expansion of the caudal portion, and has been compared with the squamosal of the Menefee skull and thus, by implication, with CPC 274. However, the Nipple Butte squamosal lacks any indication of the oblique squamosal ridge, and thus any potential identity is rejected. Based on hypotheses of dinosaur endemism [17–19], it is likely that the southern centrosaurines were quite different from northern ones. Sampson and Loewen [19] point out that the majority of horned dinosaurs are known only from the formation of discovery, and that species durations are considerably less than 1 million years. This makes it inadvisable to refer either the markedly earlier Menefee centrosaurine or the geographically removed Fort Crittenden centrosaurine from Arizona to CPC 274 Additionally, CPC 274 shares a character with *Diabloceratops*, *Avaceratops*, *Xenoceratops*, *and Nasutoceratops* that is not found in more derived centrosaurines, the lack of imbricated undulations on the parietal frill [46]. The squamosal of CPC 274 has undulations, but the fragment of parietal that has the parietal edge and epoccipital preserved clearly does not have another one flanking it on either side and in fact has an embayment on either side of the undulation wider than the epoccipital. This could potentially be a juvenile characteristic in *Avaceratops*, but the clear late subadult to adult stage of CPC 274 based on vertebral suture closure precludes this for this specimen. Considering that this character is definitively present in a different centrosaurine supports that the feature is real in *Avaceratops* as well. Future finds will hopefully better decipher this character for CPC 274 as it is unclear which epoccipitals are preserved on the parietal of CPC 274, which makes it difficult to compare presence of a lack of imbricated undulations with other better known taxa.

It is a widely held view that the squamosal in ceratopsids is diagnostic only at the subfamily [54]; this element clearly separates centrosaurines from chasmosaurines [55], but within subfamilies this bone tends to be highly conserved. Within Chasmosaurinae several taxa have been defined recently on the basis of the squamosal alone: *Ojoceratops fowleri* [56], *Mercuriceratops gemini* [57]. Geometric morphometric analysis has successfully separated *Triceratops* squamosals from those of *Torosaurus* [58]. A limitation of the geometric morphometric study cited is that only outlines not internal features of the squamosal are accessible, and so the presence of a ridge and rugose nob on the CPC 274 squamosal does not register on such an analysis. Within Centrosaurinae, *Avaceratops*, *Spinops*, and *Diabloceratops* have taxonomically distinctive squamosals. Thus, it seems premature to rule out the use of the squamosal as an apomorphic element in ceratopsid phylogenetic studies. It may be possible to separate northern and southern centrosaurines using the squamosal given the similiarities between CPC 274, the Menefee centrosaurine, the Nipple Butte skull, and *Nasutoceratops*.

### Biogeography and Late Cretaceous Endemism in the Western Interior

The formation of Laramidia during the Cretaceous with the opening of the Western Interior Seaway and the large number of dinosaurs preserved during the latest Cretaceous (Campanian and Maastrichtian) has provided an excellent window in time for workers to study dinosaur biogeography and provincialism. The picture that has appeared from this excellent dataset is that many dinosaurs had surprisingly small ranges and were largely endemic, at least with distinct northern and southern faunas [17–19]. Due to better preservation of fossils, a greater abundance of fossils, and more people working in the northern region of the western United States and Canada, the number of dinosaurs from northern Laramidia is much higher than from southern Laramidia. Using centrosaurines as an example, 13 of 15 genera from the Western Interior are from north of Colorado (Fig 1). Among diagnosable centrosaurines, only *Diabloceratops* and *Nasutoceratops* [20, 21] are known from southern Laramidia (Fig 1). However, this is not to say that centrosaurine material (and that of other dinosaurs) is not found in the southern Western Interior. A partial centrosaurine that preserved much of the skull and skeleton is known from New Mexico [22] and the first centrosaurine material from Mexico was found in the 1950s [2]. However, none of this material was diagnosable despite that evidence suggests that southern Laramidia had a unique fauna distinct from the north [1, 15, 17, 18, 24]. Therefore, it is likely that cladistic studies of North American Late Cretaceous groups are missing critical information only found in animals from southern Laramidia.

Centrosaurines appear to have been common in the south even if their skeletons are not well preserved. CPC 279 from the Cerro del Pueblo Formation of the Parras Basin represents the southernmost known extent of the centrosaurine range [1]. They are also known from the Aguja, Menefee, Wahweap, Kaiparowits, and Fort Crittenden Formations with many other dinosaurs demonstrating that southern Laramidia had a diverse, extensive, but understudied Late Cretaceous ecosystem [59–62]. Now CPC 274 offers yet another tantalizing, yet presently undiagnosable, southern centrosaurine specimen. More exploration in southern Western Interior rocks will undoubtedly produce a greater understanding of the fauna of Laramidia as a whole as well as the phylogenetic relationships between many Late Cretaceous groups.

## Conclusions

We present a new, but as of yet undiagnosable, southern centrosaurine from Mexico, CPC 274. CPC 274 is a basal centrosaurine grouping with *Avaceratops* and *Nasutoceratops*. Although centrosaurines have been known from southern Laramidia for many years, they were not included in cladistic analyses until 2010 due to the paucity of material until the discovery and description of *Diabloceratops* [20]. Considering that Late Cretaceous dinosaurs were likely endemic to northern and southern Laramidia [17, 18], a lack of southern Laramidian taxa in cladistic analyses is likely skewing phylogenies. Therefore, it is desirable to redouble efforts to discover new dinosaurs from southern Laramidia in order to rectify this issue. With the promise of the Cerro del Pueblo Formation and now the Aguja Formation in Mexico, as well as more northern Campanian age rocks, such as the Menefee Formation [61, 62], the skew towards northern taxa will hopefully diminish in the future. As with all paleontological studies, the need for new specimens is paramount to gaining a greater understanding of phylogeny.

## Supporting Information

S1 Multimedia The majority of elements that were found with CPC 274 were surface scanned using a Polhemus FastSCAN system in order to allow readers the ability to manipulate the bones in a 3D environment to assess features directly. The files are .obj files and can be visualized in MeshLab^™^, which can be downloaded for free (MeshLab, Visual Computing Lab—ISTI—CNR http://meshlab.sourceforge.net/). Surface scan of the squamosal.
(OBJ)

S2 Multimedia Surface scan of the parietal fragment 1. (OBJ)

S3 Multimedia Surface scan of the parietal fragment 2. (OBJ)

S4 Multimedia Surface scan of the premaxilla. (OBJ)

S5 Multimedia Surface scan of the dentary. (OBJ)

S6 Multimedia Surface scan of the scapula. (OBJ)

S7 Multimedia Surface scan of the femur. (OBJ)

S8 Multimedia Surface scan of the dorsal vertebra. (OBJ)

S1 Table Phylogeny information. Character codings for the matrix based on Evans and Ryan (2015). The dataset was assembled in Microsoft Excel (Office Professional 2008) and was analyzed in TNT v. 1.1 (Goloboff et al., 2008). The tree search was done using the traditional search option with the tree bisection and reconnection swapping algorithm set at 10,000 random addition sequence replicates and 10 random seeds. A single most parsimonious tree was found and is reported in the manuscript (Fig 13). Bootstrapping was done using the STATS.RUN script in TNT.
(XLSX)

## Abbreviations

ANS: Academy of Natural Science of Drexel University, Philadelphia, PA, USA
CPC: Colección Paleontológica de Coahuila, Saltillo, Coahuila, Mexico
NMMNH: New Mexico Museum of Natural History, Albuquerque, New Mexico, USA
MOR: Museum of the Rockies, Bozeman, Montana, USA
